# Work–family conflict and burnout in night-shift nurses with children: the mediating roles of recovery experience and fatigue

**DOI:** 10.3389/fpubh.2026.1937541

**Published:** 2026-09-08

**Authors:** Ting Dong, Guangke Wang, Lingcao Ma, Shuying Chang, Xiaolin Song, Hanqing Dai, Kunhua Hou

**Affiliations:** 1 Department of Otolaryngology Head and Neck Surgery, Henan Provincial Intelligent Nursing and Transformation Engineering Research Center, Henan Provincial Key Medicine Laboratory of Nursing, Henan Provincial People’s Hospital, Henan University People’s Hospital, Zhengzhou, Henan, China; 2 Department of Neurology, Henan Provincial Intelligent Nursing and Transformation Engineering Research Center, Henan Provincial Key Medicine Laboratory of Nursing, Henan Provincial People’s Hospital, Henan University People’s Hospital, Zhengzhou, Henan, China

**Keywords:** burnout, children, fatigue, night-shift nurses, recovery experience, work–family conflict

## Abstract

**Background:**

Burnout among nurses is a major global concern, particularly among those working night shifts, which can compromise the quality of nursing care, increase turnover intentions, and threaten the stability of the nursing workforce. Previous studies have shown that work–family conflict correlates positively with burnout in nurses, but not enough research has been performed on its relationship among night-shift nurses with children. In addition, the mechanism of this relationship is not completely clear.

**Objective:**

This study aimed to examine the relationships among work–family conflict, recovery experience, fatigue, and burnout among night-shift nurses with children, with particularly emphasis on the mediating roles of recovery experience and fatigue.

**Methods:**

A multicenter cross- sectional survey was conducted between November and December 2025 among night-shift nurses with children recruited from four tertiary Grade A hospitals in Henan Province, China, using convenience sampling method. Participants completed an anonymous online questionnaire that included measurement of work–family conflict, recovery experience, fatigue, and burnout. Pearson correlation analyses were performed to examine the relationships among the study variables. Mediation effects were tested using Hayes’ PROCESS macro (model 6).

**Results:**

A total of 305 night-shift nurses with children participated in the study. Work–family conflict was positively associated with fatigue and all three dimensions of burnout, including emotional exhaustion, depersonalization, and reduced personal accomplishment. Recovery experience was negatively associated with fatigue and all three dimensions of burnout. Mediational analyses revealed that work–family conflict was significantly associated with burnout, particularly emotional exhaustion and reduced personal accomplishment. Both recovery experience and fatigue independently mediated the relationship between work–family conflict and burnout. Moreover, a notable sequential mediation pathway was identified, indicating that work–family conflict is positively correlated with burnout. This relationship is sequentially associated with a diminished recovery experience and increased fatigue.

**Conclusion:**

Work–family conflict is positively associated with burnout among night-shift nurses with children, both directly and indirectly via lower recovery experience and higher fatigue. Recovery experience and fatigue mediate this association both independently and sequentially. Given these associations, interventions aimed at promoting recovery experiences and mitigating fatigue may serve as potential strategies for alleviating burnout among night-shift nurses with childcare responsibilities.

## 1 Introduction

Night shift work can have substantial adverse effects on nurses’ physical and mental health. Previous studies have demonstrated that night-shift work schedule disrupt circadian rhythms which leads to sleep deprivation, impaired cognitive performance, and reduced work efficiency (1). Furthermore, shift work and nonstandard working hours often interfere with family life, social participation, and leisure activities, which undermining employees’ work-life balance. Among healthcare professionals, nurses are particularly vulnerable to work–family conflict, and its prevalence has continued to increase in recent years (2). A study in Mosul shows that a majority (61%) of nurses reported poor work-life balance, with only 7.5% achieving an excellent level (3). In China, approximately 50% of nurses report experiencing persistent or chronic work–family conflict (4). Studies suggests that parenthood, particularly caring for young children, is associated with higher levels of work–family conflict (5, 6). Work-life balance is a critical component of nurses’ well-being. However, night-shift nurses with children must not only handle a heavy clinical workload but also juggle a multitude of family responsibilities, including caring for their children and managing household chores. This dual burden may exacerbate work–family conflict, which results in elevated psychological stress and a greater susceptibility to burnout (1, 7).

Burnout has been recognized by the World Health Organization as an occupational phenomenon and represents a major threat to the mental health of healthcare professionals worldwide (8). Among healthcare workers, nurses consistently report some of the highest levels of burnout, with prevalence estimates among clinical nurses in China reaching nearly 60% (9, 10). Burnout is particularly prevalent among night-shift nurses, who frequently experience prolonged working hours, circadian rhythm disruption, and excessive workloads (8, 11). The consequences of burnout are substantial, burnout may reduce nurses’ work engagement, compromise the quality and safety of patient care, diminish professional fulfillment and subjective well-being, and increase turnover intentions (12–14). Ultimately, these outcomes contribute to the ongoing shortage of nursing personnel and threaten the sustainability of the healthcare workforce. Studies have consistently demonstrated a positive association between work–family conflict and burnout among nurses, which indicates that greater work–family conflict is associated with more severe burnout symptoms (15–17). Family responsibilities, particularly childcare, have been identified as important contributors to burnout among female nurses (18). Despite growing evidence linking work–family conflict to burnout, research focusing specifically on night-shift nurses with children remains limited.

Recovery experience refers to the psychological process through which individuals restore depleted physical and mental resources following exposure to work-related stress. It encompasses four dimensions: psychological detachment, relaxation, mastery, and control experiences (19, 20). Recovery experiences play a critical role in alleviating work-related fatigue and stress and facilitating the replenishment of personal resources. Among the interventions proposed to occupational burnout, physical activity and recovery experiences have been identified as particularly effective. Previous research has demonstrated that physical activity is negatively associated with nurses’ burnout, and that psychological detachment, relaxation, and mastery experiences mediate the relationship between physical activity and burnout (21). The fundamental function of recovery experience is to facilitate recovery from work-related strain and replenish depleted resources, which is positively correlated with lower burnout risk. However, it remains unclear whether recovery experience serves as a protective factor that weakens the adverse association between work–family conflict and burnout among night-shift nurses with children.

Fatigue is a critical concern among nurses, particularly those working night shifts. Previous studies have consistently reported that night-shift nurses experience higher levels of fatigue and poorer sleep quality than their day-shift counterparts (22). Work–family conflict has been shown to increase psychological strain, thereby contributing to greater fatigue (23). Fatigue not only directly affect burnout but may also impact burnout indirectly through its influence on factors such as psychological capital and social support (6, 24). Although fatigue has been recognized as an important predictor of burnout, research examining its mediating role in the relationship between work–family conflict and burnout among night-shift nurses with children remains limited. Furthermore, no previous study has simultaneously examined recovery experience and fatigue as sequential mediators linking work–family conflict to burnout in this population.

In summary, the relationships among work–family conflict, recovery experience, fatigue, and burnout among night-shift nurses with children remain insufficiently understood. In particular, the specific mechanisms linking work–family conflict to burnout require further study. Therefore, this study aimed to examine the associations among these four factors, as well as the mediating roles of recovery experience and fatigue in the relationship between work–family conflict and burnout. By clarifying these mechanisms, this study seeks to provide both theoretical evidence and practical implications for the development of targeted interventions to reduce burnout among night-shift nurses with childcare responsibilities.

This study is grounded in the Conservation of Resources Theory (25), which posits that individuals strive to acquire, maintain, and protect valued resources, with psychological stress emerging when these resources are threatened or diminished. For night-shift nurses with children, work–family conflict may affect their limited personal resources, including time, energy, and emotional capacity. According to the Resource of Conservation Theory, when resources are depleted, individuals must replenish them to prevent further loss. The recovery process may play a key role in this resource rebuilding process, when the recovery process is significantly impeded by work–family conflict, the inability to restore depleted resources may be linked to the onset of fatigue, which is subsequently correlated with burnout. Based on the existing literature and theoretical framework, the following hypotheses were proposed:

*H*1. Work–family conflict is positively associated with burnout and fatigue.

*H*2. Work–family conflict is negatively associated with recovery experience.

*H*3. Recovery experience is negatively associated with burnout and fatigue.

*H*4. Fatigue is positively associated with burnout.

*H*5. Recovery experience and fatigue independently and sequentially mediate the relationship between work–family conflict and burnout.

## 2 Materials and methods

### 2.1 Study design and participants

The multicenter and cross-sectional study was conducted in four tertiary comprehensive hospitals located in two prefecture-level cities in Henan Province, China. Participants were recruited using a convenience sampling strategy.

The inclusion criteria were as follows: (a) registered nurses with ≥1 year of clinical nursing experience; (b) nurses who had been working rotating night shifts for≥6 months and were currently engaged in night-shift work; (c) nurses who were raising one or more children; and (d) nurses who voluntarily agreed to participate and provided informed consent. The exclusion criteria included: (a) nurses who were on leave or otherwise absent from work during the study period; and (b) nursing intern.

The sample size was determined utilizing G*Power version 3.1.9.4. The parameters specified included a statistical power of 0.95, a significance level of 0.05, seven predictors, and an effect size of 0.15, resulting in a minimum required sample size of 154 participants. To accommodate an anticipated dropout rate of 20%, the sample size was adjusted to 185 participants. Invalid questionnaires were defined as those with more than 10% inconsistent responses or those in which the same response option was selected for more than 10 consecutive items (11). In this case, a total of 310 questionnaires were distributed. After excluding five invalid questionnaires, 305 valid responses were retained for analysis which yields an effective response rate of 98.39%.

### 2.2 Measures

#### 2.2.1 Demographic Questionnaire

A self-designed demographic questionnaire was developed by the research team to collect participants’ demographic and occupational characteristics. The questionnaire included information on age, years of nursing experience, education level, professional nursing titles, number of children, duration of night-shift work, and the number of rest days following night shifts.

#### 2.2.2 Work–Family Conflict Scale

The Work–Family Conflict Scale (WAFCS) was developed to assess work–family conflict among employed parents (26). The scale consists of 10 items divided into two dimensions: work-to-family conflict (WFC, five items) and family-to-work conflict (FWC, five items). Responses are rated on a seven-point Likert scale ranging from 1 (“strongly disagree”) to 7 (“strongly agree”). Higher scores indicate greater levels of work–family conflict. The Chinese version of the scale has demonstrated satisfactory reliability and validity (27). In the present study, Cronbach’s alpha coefficient for the scale was 0.919 which indicates excellent internal consistency.

#### 2.2.3 Maslach Burnout Inventory-General Survey

The Maslach Burnout Inventory–General Survey (MBI–GS) is a self-assessment tool developed by Christina Maslach and colleagues to assess occupational burnout (28). The Chinese version of the MBI-GS was adapted by Li Cand has demonstrated satisfactory reliability and validity among Chinese healthcare professionals (29). The scale comprises 15 items across three dimensions: emotional exhaustion, depersonalization, and reduced personal accomplishment. Items are rated on a seven-point Likert scale ranging from 0 (“never”) to 6 (“very frequently”). Reduced personal accomplishment is reverse scored. Higher score across all three dimensions indicates higher levels of burnout. Cronbach’s alpha coefficient in this study was 0.963 which indicates excellent internal consistency.

#### 2.2.4 Recovery Experience Questionnaire

Recovery experience was assessed using the Recovery Experience Questionnaire developed by Sonnentag and translated into Chinese by Zhang RR (19, 30). The questionnaire consists of 16 items covering four dimensions: mastery experience, psychological detachment, control experience, and relaxation experience. Responses are rated on a five-point Likert scale ranging from 1 (“not at all”) to 5 (“completely”) which reflects participants ‘experiences during non-work time. Higher scores indicate greater levels of recovery experience. In this study, Cronbach’s alpha coefficient was 0.928 which illustrates excellent internal consistency.

#### 2.2.5 Brief Fatigue Inventory

The Brief Fatigue Inventory Scale (BFI) (31) was used to assess the degree of fatigue. The scale contains nine items to assess the severity of fatigue and its interference with daily functions, including general activities, mood, walking ability, work, interpersonal relationships and enjoyment of life. Each item was scored from 0 to 10, with higher scores indicating more severe fatigue and functional impairment. In this study, the Cronbach’s α coefficient was 0.955, indicating that the scale has excellent internal consistency.

### 2.3 Data collection procedures

Data collection was conducted from November 27 to December 31, 2025, using a widely used online survey platform in China—the Questionnaire Survey Platform.^1^ Prior to data collection, the research team contacted the head nurses of participating hospitals, explained the research objectives and procedures, and obtained their consent. Before completing the questionnaire, participants thoroughly understood the research objectives, the principle of voluntary participation, confidentiality measures, and data protection measures. All participants signed informed consent. The questionnaire link and QR code were sent via WeChat group. To prevent duplicate responses, each IP address was only allowed to submit the questionnaire once. All questionnaire items were mandatory; incomplete questionnaires would not be submitted.

### 2.4 Ethical considerations

This study was approved by the Ethics Committee of Henan University (Approval No. HUSOM2021-265). All participants provided informed consent before participation. Participants were informed that their response would be used solely for research purposes and that all information would remain anonymous and confidential. The study was conducted in accordance with ethical principles of Declaration of Helsinki.

### 2.5 Data analysis

Data were analyzed using IBM SPSS Statistics version 27.0 (IBM Corp., Armonk, NY, USA). Categorical variables were summarized as frequencies and percentages, whereas continuous variables were presented as arithmetic means and standard deviations (SDs).

Common method bias (CMB) was assessed using Harman’s single-factor test (32). A first-factor variance contribution rate below 40% was considered indicative of an acceptable level of common method bias. In addition, we employed a full collinearity test method to provide supplementary validation.

Mediation analyses were conducted using Hayes’ PROCESS macro (model 6) (33) to examine the independent and mediating effects of recovery experiences and fatigue. The statistical significance was determined using 5,000 bootstrap resamples and bias-corrected 95% confidence intervals. A mediation effect was considered statistically significant when the corresponding confidence interval did not include zero. Based on previous recommendations, effect sizes were interpreted as large (*β* > 0.30), moderate (0.10 ≤ β ≤ 0.30), and small (*β* < 0.10) (34). Age, years of nursing experience, educational level, number of children, duration of night-shift work, and number of rest days following night shifts were included as covariates in the mediation models.

## 3 Results

### 3.1 Participant characteristics

The participants ranged in age from 26 to 57 years, with nursing experience ranging from 3 to 37 years. Most participants held a bachelor’s degree (82.3%), were nurse-in-charge level staff (77.1%), and had one child (51.5%). Detailed demographic characteristics are presented in Table 1.

**Table 1:** Demographic characteristics of participants (*N* = 305).

Variable	Number
Age, years	35.81 ± 4.50
Nursing experience, years	13.14 ± 5.10
Education level	
Junior college	27(8.9%)
Bachelor degree	251(82.3%)
Master or above	27(8.9%)
Professional nursing titles	
Nurse	10(3.3%)
Senior nurse	56(18.4%)
Nurse-in-charge	235(77.0%)
Associate chief nurse	4(1.3%)
Number of children	
One	157(51.5%)
Two	144(47.2%)
Three	4(1.3%)
Duration of night-shift work, hours	12.15 ± 2.26
The number of rest days following night shifts	
One day	33(10.8%)
Two days	60(19.7%)
1 or 2 days; mostly 1 day	103(33.8%)
1 or 2 days; mostly 2 day	109(35.7%)

### 3.2 Common method bias analysis

Our analysis demonstrated that no single or predominant factor explained the majority of the variance in the exploratory factor analysis, specifically, eight common factors exhibited eigenvalues exceeding 1. Notably, the first factor accounted for 37.37% of the variance, which is below the 40% threshold. In addition, we additionally employed the full collinearity test approach, a series of regressions were conducted where each latent construct was regressed on all other constructs, the resulting full collinearity Variance Inflation Factor (VIF) values are all less than 3. While these results do not definitively eliminate the possibility of common method bias (CMB), they provide evidence suggesting that CMB may not pose a significant threat to the validity of our findings.

### 3.3 Correlation analysis

Descriptive statistics and Pearson correlation coefficients for all study variables are presented in Table 2. Emotional exhaustion, depersonalization, and reduced personal accomplishment were positively correlated with work–family conflict and fatigue and negatively correlated with recovery experience. In addition, work–family conflict was positively associated with fatigue and negatively associated with recovery experience. Recovery experience was negatively correlated with fatigue.

**Table 2:** Descriptive statistics and bivariate correlations of study measures.

Measurer	Mean	SD	1		2		3		(1)		(2)		(3)
			r	95%CI	r	95%CI	r	95%CI	r	95%CI	r	95%CI	
1.Work family conflict	40.61	12.24	1										
2.Recovery experience	49.33	11.80	−0.328**	[−0.424, −0.224]	1								
3.Fatigue	5.25	2.49	0.492**	[0.402, 0.573]	−0.369**	[−0.462, −0.267]	1						
4.Occupational burnout													
(1)Emotional exhaustion	15.57	8.14	0.520**	[0.433, 0.598]	−0.389**	[−0.480, −0.289]	0.608**	[0.532, 0.674]	1				
(2)Depersonalization	8.59	7.26	0.492**	[0.402, 0.573]	−0.404**	[−0.494, −0.306]	0.479**	[0.388, 0.562]	0.767**	[0.716, 0.809]	1		
(3)Reduced personal accomplishment	8.09	5.12	0.457**	[0.363, 0.541]	−0.411**	[−0.500, −0.313]	0.389**	[0.289, 0.480]	0.569**	[0.488, 0.640]	0.771**	[0.721, 0.813]	1

**p <0.01.

### 3.4 Mediation analysis

**Figure 1:**
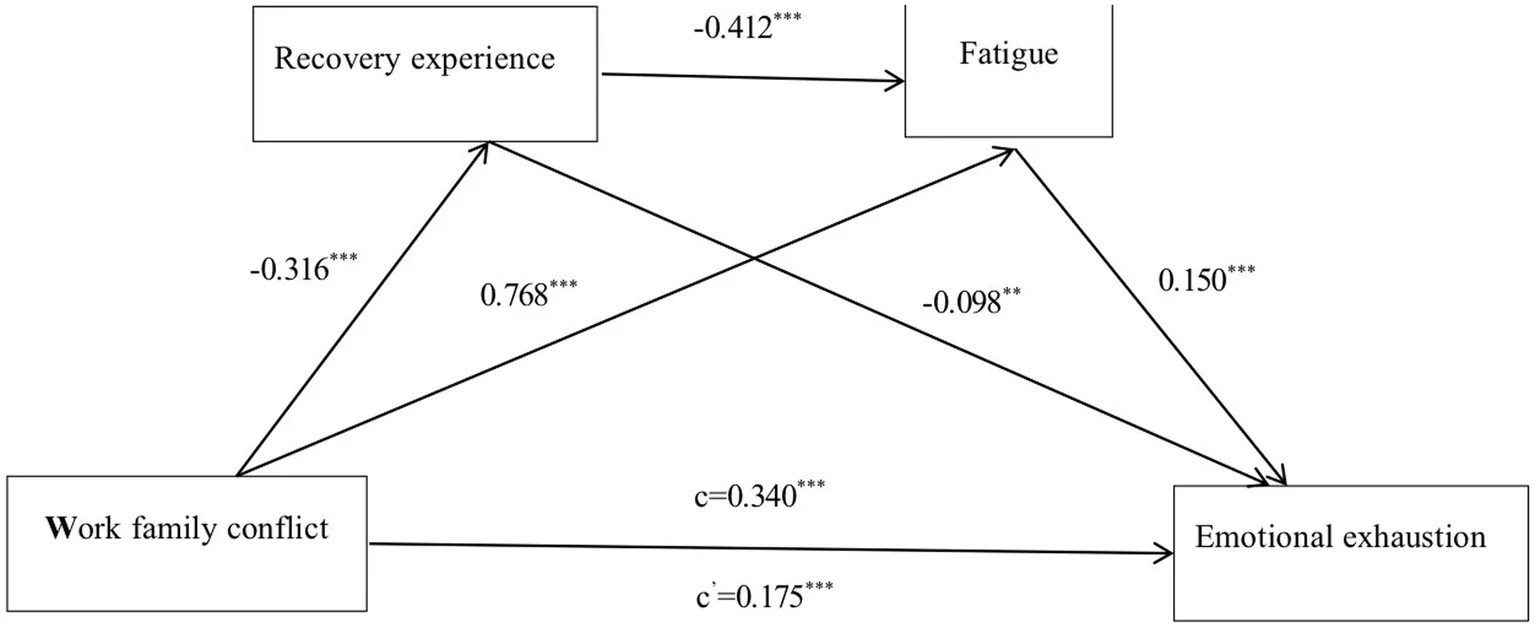
The mediating effects on work–family conflict and emotional exhaustion. Standardized regression coefficients were displayed in the path diagram; *c* = total effect; *c’* = direct effect; ∗∗∗*p* < 0.001, ∗∗*p* < 0.01.

**Figure 2:**
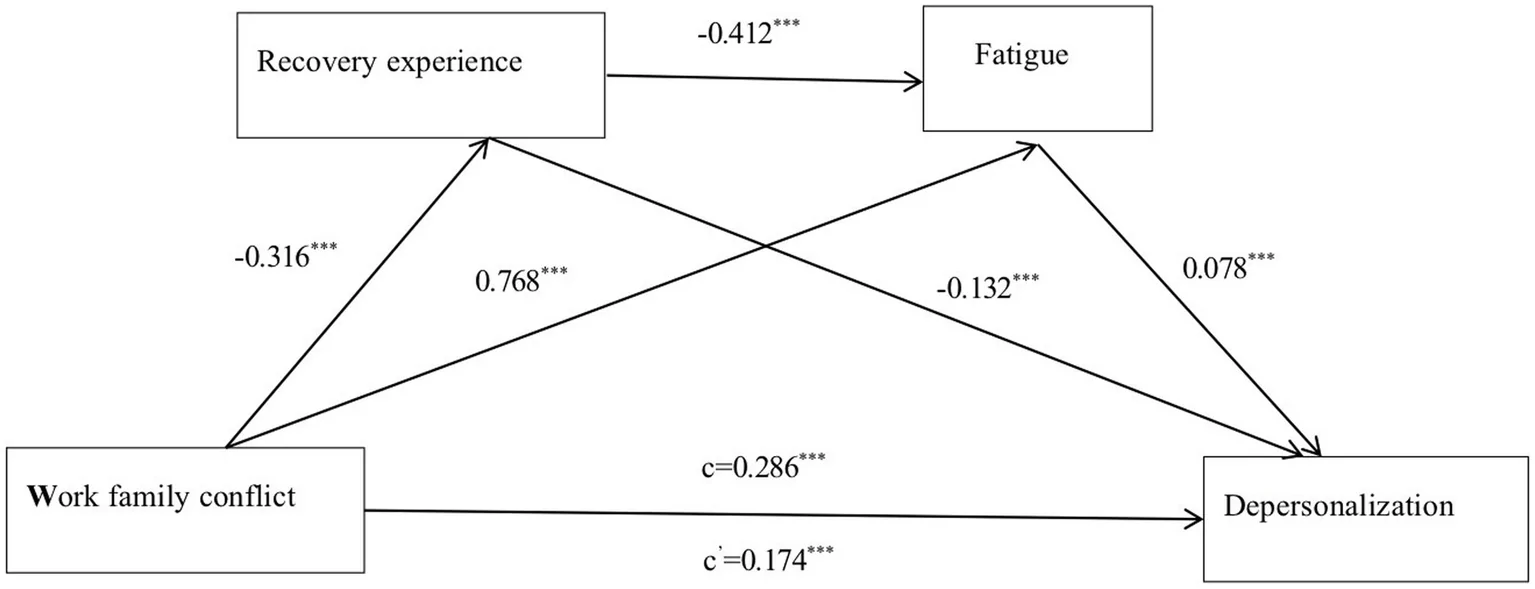
The mediating effects on work family conflict and depersonalization. Standardized regression coefficients were displayed in the path diagram; *c* = total effect; *c’* = direct effect; ∗∗∗*p* < 0.001.

**Figure 3:**
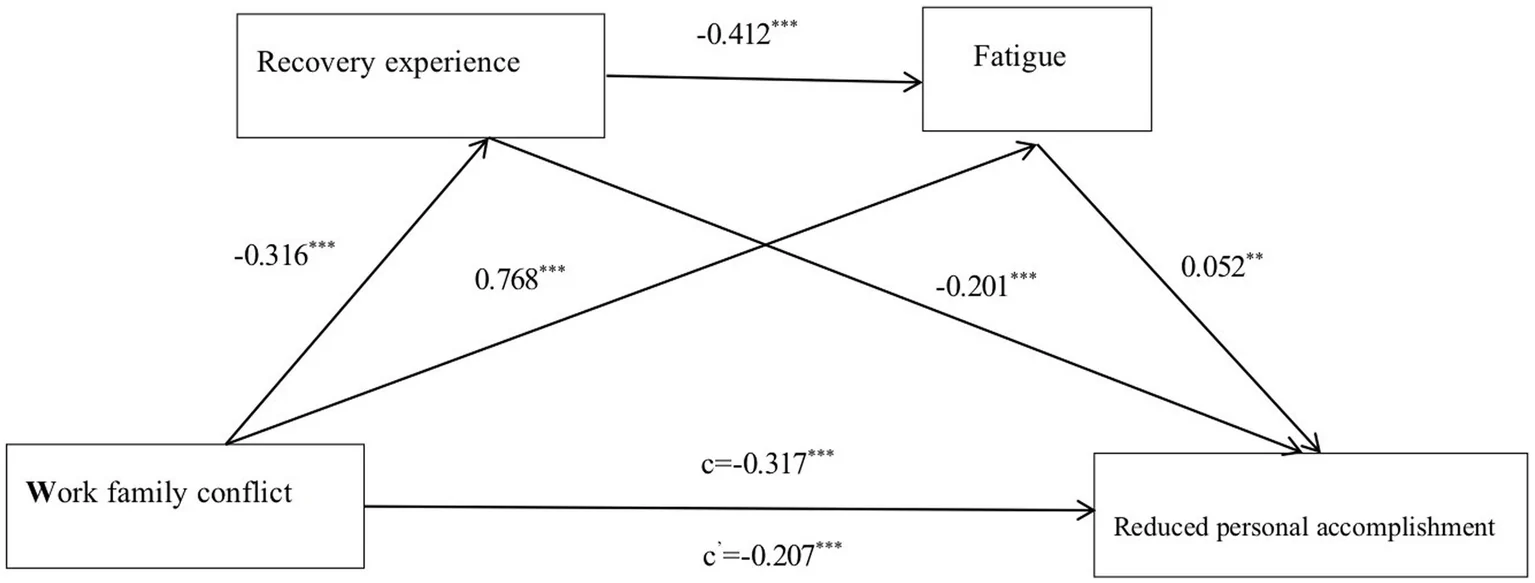
The mediating effects on work family conflict and reduced personal accomplishment. Standardized regression coefficients were displayed in the path diagram; *c* = total effect; *c*′ = direct effect; ∗∗∗*p* < 0.001, ∗∗*p* < 0.01.

Before performing the multiple mediation analysis, the essential statistical assumptions were thoroughly evaluated. The assumption of linearity was confirmed through the visual inspection of scatterplots. Multicollinearity was assessed using the Variance Inflation Factor (VIF) and Tolerance, which indicated no significant multicollinearity among the variables. The results of the mediation analyses are presented in Table 3 and Figures 1–3. Work–family conflict was significantly associated with emotional exhaustion, depersonalization, and reduced personal accomplishment. After controlling for recovery experience and fatigue, the direct effects of work–family conflict on all three burnout dimensions remained statistically significant.

**Table 3:** Total, direct, and indirect effects of the mediation model.

Effect	Effect size	Bootstrap SE	Bootstrap 95% CI
Emotional exhaustion			
Total effect (work family conflict →emotional exhaustion)	0.340	0.033	[0.276, 0.404]
Direct effect (work family conflict →emotional exhaustion)	0.175	0.034	[0.109, 0.241]
Indirect effect	0.165	0.028	[0.115, 0.222]
Work family conflict →recovery experience →emotional exhaustion	0.031	0.013	[0.008, 0.058]
Work family conflict →fatigue →emotional exhaustion	0.115	0.025	[0.072, 0.168]
Work family conflict →recovery experience →fatigue →emotional exhaustion	0.020	0.007	[0.007, 0.035]
Depersonalization			
Total effect (work family conflict →depersonalization)	0.286	0.029	[0.228, 0.344]
Direct effect (work family conflict →depersonalization)	0.174	0.032	[0.111, 0.237]
Indirect effect	0.112	0.026	[0.066, 0.169]
Work family conflict →recovery experience →depersonalization	0.042	0.016	[0.016, 0.076]
Work family conflict →fatigue →depersonalization	0.060	0.017	[0.031, 0.099]
Work family conflict →recovery experience →fatigue →depersonalization	0.010	0.004	[0.003, 0.020]
Reduced personal accomplishment			
Total effect(work family conflict →reduced personal accomplishment)	0.317	0.036	[0.245, 0.388]
Direct effect(work family conflict →reduced personal accomplishment)	0.207	0.040	[0.128, 0.286]
Indirect effect	0.110	0.029	[0.054, 0.168]
Work family conflict →recovery experience →reduced personal accomplishment	0.063	0.019	[0.029, 0.104]
Work family conflict →fatigue → reduced personal accomplishment	0.040	0.019	[0.003, 0.078]
Work family conflict →recovery experience →fatigue →reduced personal accomplishment	0.007	0.004	[0.001, 0.016]

Standardized coefficients were used to estimate the effect sizes. CI, confidence interval; SE, standard error.

Recovery experience played as a significant mediator in the relationships between work–family conflict and emotional exhaustion, depersonalization, and reduced personal accomplishment. Fatigue also served as a significant independent mediator in these relationships. Furthermore, the serial mediation pathway involving recovery experience and fatigue was statistically significant across all three burnout dimensions.

Specifically, higher levels of work–family conflict were related to lower levels of recovery experience, which is associated with greater fatigue as well as higher levels of emotional exhaustion, depersonalization, and reduced personal accomplishment. These results support both the independent mediating effects of recovery experience and fatigue as well as a sequence in the same between work–family conflict and burnout.

## 4 Discussion

This study examined the relationships among work–family conflict, recovery experience, fatigue, and burnout among night-shift nurses with children. According to our research, night-shift nurses with childcare responsibilities reported relatively high levels of work–family conflict and burnout, particularly emotional exhaustion and depersonalization (11, 35). Given the unique challenges associated with balancing demanding clinical responsibilities and family caregiving obligations, understanding the mechanisms underlying burnout in this population is of considerable importance. Our study demonstrated a significant positive association between work–family conflict and burnout, recovery experience and fatigue were identified as both independent and sequential mediators of this relationship. In other words, work–family conflict may impair post-work recovery, is associated with increased fatigue, and ultimately has a positive effect on burnout. In general, these findings provide new insights into the mechanisms of burnout for night shift nurses who have children and also identify potential areas where interventions can be developed.

First, work–family conflict was significantly associated with higher levels of burnout among night-shift nurses with children. Specifically, the strongest relationships were seen between work–family conflict and emotional exhaustion and decreased feelings of personal accomplishment. This finding is consistent with earlier studies indicating that work–family conflict is a strong predictor of burnout in nurses (36–38). The present findings further suggest that childcare responsibilities may exacerbate the adverse effects of work–family conflict on occupational well-being. Previous studies have shown that primary care responsibility within family and childcare are identified as an important factor that affect nurses’ burnout (18, 39). Many nurses experience increased work–family conflicts after the birth of children due to changes in family dynamics and increased distractions, which potentially lead to elevated stress levels among nurses who are also parents (17, 40). Other studies found that nurses who have a lot of family responsibilities are more likely to experience burnout and may be more inclined to consider leaving the profession (41).

This issue may be particularly prominent in the context of Chinese culture. Mothers remain the primary caregivers for infants aged 0–3 years (42), with 74.1% of children primary cared for by their mothers at night (43). Consequently, night-shift nurses with children must simultaneously manage heavy professional responsibilities and nursing duties, which exposes them to a higher risk of role overload and burnout. From a practical perspective, the study highlights the importance of organizational and social support, a supportive work environment, flexible scheduling and effective coping strategies may help mitigate the negative impact of work–family conflict on burnout (44, 45). In addition, social support from family and colleagues can serve as a valuable resource for managing conflicting role needs and alleviating psychological stress (46). Therefore, head nurses should take family circumstances into account when developing shift schedules and should implement family-friendly workplace policies whenever possible (47). On an individual level, encouraging family members to share more childcare and housework responsibilities may help reduce role conflict and alleviate the stress of night shift nurses with children (48).

It is important to acknowledge that variations in organizational culture, healthcare contexts and child-rearing policies necessitate a cautious interpretation of the findings of this study. In low and middle-income nations, exemplified by Iraq, challenges related to work–life balance may be exacerbated by restricted healthcare resources, substantial patient volumes and limited organizational support (49–51). In post-conflict settings like Mosul City, women frequently assume primary caregiving roles, and the concurrent pressures of professional and domestic responsibilities can exacerbate work–life imbalance (52). In addition, childcare policies and social support systems exhibit considerable variation across countries. In China, the absence of universal, state-subsidized childcare for children under three compels working mothers—particularly those working irregular night shifts—to rely heavily on informal family networks, primarily grandparent care, or expensive private childcare options. This structural inadequacy may disproportionately exacerbate work–family conflict and burnout when compared to countries that have established comprehensive, state-funded childcare systems and more flexible parental leave policies. Therefore, understanding the relationship between work–family conflict and burnout within the context of local culture and healthcare is crucial for designing effective organizational and family-supportive interventions.

Second, our research has found that recovery experiences mediate the relationship between work–family conflict and burnout. This is consistent with previous findings that recovery experiences are negatively correlated with burnout (19, 53, 54). A meta-analysis by Bennett and colleagues illustrated that recovery experiences can reduce burnout and enhance individuals’ sense of energy (55). Specifically, psychological disengagement helps restore depleted physical and mental resources and reduces emotional exhaustion (56). Individuals who are able to fully detach from work during their off-work hours are more likely to recover effectively from occupational stress and maintain mental health (21). Work–family conflict may prolong exposure to work-related and family-related stressors, thereby impairing resilience. Previous studies have shown that both work-family and family–work conflict are associated with emotional exhaustion and depressive symptoms (57). When individuals are cognitively and emotionally preoccupied with unresolved role demands, the opportunity for effective recovery is reduced. Therefore, insufficient recovery may hinder resource replenishment and increase the risk of burnout. The results of this study suggest that recovery experience is an important psychological mechanism connecting work–family conflict and burnout, and therefore may be an important target for intervention. Encouraging physical activity, leisure engagement and psychological detachment during non-work time may facilitate recovery and reduce burnout risk (21). In addition, the implementation of recovery-oriented work design and flexible scheduling is instrumental in mitigating work–family conflict and facilitating recovery, for example, implementing flexible or self-scheduling systems that allow nurses with childcare responsibilities to more accurately predict and effectively manage their shifts. Furthermore, the strict enforcement of adequate rest periods between consecutive night shifts (such as avoid less than 11 h, 2 days off after night shift, forward-shifting rotation) is essential (58), as it aids nurses in achieving both physical and mental disengagement from work. At the individual level, hospitals should implement evidence-based resilience interventions to assist nurses in managing the physiological and psychological challenges associated with night shifts, for instance, cognitive behavioral therapy has been shown to alleviate work–family conflict and reduce burnout (59), and can empower nurses to improve their psychological recovery experiences and strengthen their resilience against burnout. The subjective enhancement in well-being associated with restorative yoga indicates that it may have a beneficial effect on psychological health, providing an accessible and self-directed approach for managing occupational stress (60).

Third, fatigue is a significant mediating factor between work–family conflict and burnout among night shift nurses (especially those with children). Previous study has shown that shift work, especially night shifts, is associated with higher levels of physical and mental fatigue, and work–family conflict may further exacerbate this fatigue (61). Fatigue has long been considered a precursor to burnout (62), prolonged occupational stress and insufficient recovery can lead to persistent fatigue, which can gradually develop into emotional exhaustion and other core dimensions of burnout (63). These findings suggest that fatigue is an important mechanism linking work–family conflict and burnout in this population. According to the Conservation of Resource Theory, for night shift nurses who have to take on childcare responsibilities, the dual pressures of shift work and childcare may accelerate the depletion of their physiological, psychological, and cognitive resources, continuous resource depletion may manifest as fatigue (64). The fatigue accumulated from unresolved work–family conflicts transforms daily stress into the emotional exhaustion and depersonalization (65). Given the mediating role of fatigue, hospitals should adopt structured fatigue risk management programs tailored for night-shift workers. Practical strategies include napping during shift work can improve performance and decreased fatigue in shift workers (66), providing strategic caffeine and nutritional guidance helps combat night shift fatigue and maintain alertness (67), and conducting brief pre-shift fatigue check-ins to identify and reassign highly fatigued nurses from high-risk clinical tasks.

Finally, the results revealed a significant sequential mediation pathway involving recovery experience and fatigue. Specifically, higher levels of work–family conflict were associated with poorer recovery experience, which was related to greater fatigue, and this greater fatigue was further associated with higher levels of burnout. This finding provides empirical support for a progressive resource depletion process. Night-shift nurses with children often face severe rest restrictions, irregular working hours disrupt their circadian rhythms and reduce sleep quality (68), while childcare responsibilities further reduce their opportunities to relax and relieve psychological burdens (69), as a result, these nurses may have difficulty getting enough rest, leading to an inability to effectively relieve work and family stress (70). Insufficient recovery impairs resource replenishment and contributes to the accumulation of fatigue (71, 72). Over time, chronic fatigue may evolve into burnout as individuals’ adaptive resources become progressively depleted (65).

However, our theoretical framework supports the proposed sequential pathway, alternative mediation models remain plausible. For example, a parallel mediation model could be proposed, wherein work–family conflict is concurrently and independently associated with poorer recovery experience and greater fatigue. Additionally, a reverse sequential pathway is conceivable: severe fatigue may physically and cognitively hinder nurses from participating in active recovery experiences (e.g., lacking the energy for psychological detachment or mastery experiences), thereby affecting burnout. Future longitudinal studies are needed to compare these models and capture the dynamic relationship between recovery and fatigue over time.

Several limitations should be acknowledged. First, the cross-sectional design precludes causal inference regarding the relationships among work–family conflict, recovery experience, fatigue and burnout. Future longitudinal studies or ecological momentary assessment designs are needed to clarify temporal and causal relationships among these variables. Second, all variables were assessed using self-report measures, which may introduce common method bias (CMB) and social desirability bias. Although procedural and statistical methods (e.g., Harman’s single-factor test, full collinearity test) were used to minimize these effects, future studies should incorporate multi-source or objective measures whenever possible. Third, the findings were derived from a sample of Chinese night-shift nurses, caution should be exercised when extrapolating these results to other international contexts due to distinct differences in organizational culture, healthcare systems and childcare policies. Cross-cultural studies are needed to determine whether the observed mediation mechanisms are consistent across different populations. Finally, the present study focused primarily on recovery experience and fatigue, other individual, organizational and family-related factors may also influence the relationship between work–family conflict and burnout, and should be examined in future research.

## 5 Conclusion

This cross-sectional study examined the associations between work–family conflict and burnout among night-shift nurses with children, focusing on the potential indirect pathways involving recovery experience and fatigue. The results revealed a significant positive association between work–family conflict and burnout. Furthermore, recovery experience and fatigue were identified as significant independent and serial indirect links between these variables. While causal inferences cannot be drawn due to the cross-sectional design, these findings highlight recovery experience and fatigue as critical correlates of burnout, suggesting them as potential targets for future longitudinal or interventional studies aimed at mitigating burnout in this population.

## Data Availability

The original contributions presented in the study are included in the article/supplementary material, further inquiries can be directed to the corresponding authors.

## Footnotes

1
https://www.wjx.cn/
